# A Study of Overripe Seed Byproducts from Sun-Dried Grapes by Dispersive Raman Spectroscopy

**DOI:** 10.3390/foods10030483

**Published:** 2021-02-24

**Authors:** Francisco J. Rivero, Leonardo Ciaccheri, M. Lourdes González-Miret, Francisco J. Rodríguez-Pulido, Andrea A. Mencaglia, Francisco J. Heredia, Anna G. Mignani, Belén Gordillo

**Affiliations:** 1 Food Colour and Quality Laboratory, Facultad de Farmacia, Universidad de Sevilla, 41012 Sevilla, Spain; frivero@us.es (F.J.R.); rpulido@us.es (F.J.R.-P.); heredia@us.es (F.J.H.); bgordillo@us.es (B.G.); 2 Institute of Applied Physics “Nello Carrara” (IFAC), Area di Ricerca di Firenze, Via Madonna del Piano, 10-50019 Sesto Fiorentino, Italy; l.ciaccheri@ifac.cnr.it (L.C.); a.mencaglia@ifac.cnr.it (A.A.M.); a.g.mignani@ifac.cnr.it (A.G.M.)

**Keywords:** Raman scattering, vibrational spectroscopy, overripe seeds, postharvest sun drying, grape byproducts, phenolics

## Abstract

Overripe seeds from sun-dried grapes submitted to postharvest dehydration constitute a scarcely investigated class of vinification byproduct with limited reports on their phenolic composition and industrial applications. In this study, Raman spectroscopy was applied to characterize a selection of overripe seed byproducts from different white grapes (cv. Moscatel, cv. Pedro Ximénez and cv. Zalema) submitted to postharvest sun drying. The Raman measurements were taken using a 1064 nm excitation laser in order to mitigate the fluorescent effect and the dispersive detection scheme allowed a compactness of the optical system. Spectroscopic data were processed by a principal component analysis to reduce the dimensionality and partner recognition. The evolution of the Raman spectrum during the overripening process was compared with the phenolic composition of grape seeds, which was determined by rapid resolution liquid chromatography/mass spectrometry (RRLC/MS). A multivariate processing of the spectroscopic data allowed the classification of overripe seeds according to the grape variety and the monitoring of stages of the postharvest sun drying process.

## 1. Introduction

Every year, the wine industry produces great amounts of grape byproducts (grape pomace, seeds, skins or stems) that have important added value due to their chemical composition, especially phenolics [1].

Phenolic compounds are secondary metabolites of plants related to defensive responses against environmental (sunlight or temperatures) or biotic (pathogen attacks) factors [2], which have interesting applications for the pharmaceutical, food or cosmetic industries because of their antioxidant properties related to the positive effects on human health [3]. On the other hand, these bioactive compounds are responsible for sensory properties in plant-based foods contributing to the color, bitterness and astringency [4].

In warm climate regions of Mediterranean countries such as southern Spain, white grapes are submitted to postharvest dehydration by direct sun drying for increasing the sugar content in berries (>20° Brix) to elaborate traditional sweet wines [5]. The extreme sun conditions activate the gene expression involved in the phenolic biosynthesis and polymerization and, consequently, their progressive concentration in grape tissues such as seeds [6]. Previous studies have highlighted that the overripe seeds obtained from the pomace (resulting after pressing the dried grapes) still have an important phenolics content of mainly procyanidins and oligomeric flavanols that have antioxidant and copigmentation properties [7,8]. Their inherent richness in these chemicals together with the large yield due to its seasonal character make these byproducts one of the most relevant natural sources of phenolics for exploiting their health-promoting and sensory properties on an industrial scale [9]. In this sense, the assessment of the phenolic composition of this grape byproduct could bring an expansion of their industrial applications, involving both economic and environmental benefits.

Phenolics are usually identified and quantified by chromatographic or spectrophotometric methods, which involve time-consuming sample preparation and analysis, sample destruction and reagents wasting (that are occasionally toxic) [10]. On contrast, vibrational spectroscopy techniques are able to analyze many samples in a quick and non-destructive way (with fast and real-time measurements) and do not need reagents (with economic and environmental savings). These advantages make vibrational techniques valuable screening tools for quality control and support the conventional analytical methods [11,12].

Raman is a powerful vibrational spectroscopy technique commonly applied in agricultural products and food analysis [12,13]. It is based on the inelastic scattering of a monochromatic light that provides a molecular fingerprint of organic compounds, among others, according to their chemical bonds [14]. This non-invasive spectroscopic technique offers competitive advantages regarding other spectral techniques. It is less sensitive than near infrared (NIR) to the presence of water and its bands are usually sharper than the overtones of infrared (IR) bands [15]. In addition, Raman is very sensitive to functional and highly polarizable groups (C-C and C=C) commonly found in the polymer chain backbone and, hence, it could be a useful tool to evaluate polymerization [16].

Raman spectroscopy has been applied to authenticate and classify different foodstuff with food control purposes [13,14]. It has provided good results to determine the content of organic molecules (proteins, sugars, carbohydrates and lipids) and phenolic compounds (even individual phenolics) in different foods [13,17,18,19,20]. On the other hand, it has been demonstrated to be useful in confirming the linkage between cell wall components and phenolic extractability in grape seeds [21,22].

The Raman technique can be applied by compact and portable devices with excitation at 785 nm and dispersive detection schemes or by complex Fourier Transform Raman devices with excitation at 1064 nm [15,23]. The excitation at 785 nm can excite natural fluorescence that needs to be quenched or subtracted. Devices exciting at 1064 nm reduce the laser-induced fluorescence, showing a high spectral resolution with good wavelength accuracy. However, the Fourier Transform detection scheme is cumbersome and is not suitable for use in a portable device [13]. On the other hand, the strong fluorescence signals emitted by agricultural products is a challenge for Raman spectroscopy because they mask the characteristic Raman scattering signal, which limits the accuracy of the technique [18,23].

The aims of this study were to assess the potential of dispersive Raman spectroscopy as a fast and non-invasive tool to characterize overripe seeds obtained from sun-dried grape byproducts of different grape varieties and for monitoring the postharvest drying process in relation to changes in their phenolic composition.

## 2. Materials and Methods

### 2.1. Overripe Seeds from Sun-Dried Grape Byproducts

The study includes overripe seeds derived from byproducts of three varieties of *Vitis vinifera* L. white grapes submitted to postharvest dehydration in southern Spain: cv. Pedro Ximénez (PX) (Montilla-Moriles D.O., Córdoba), cv. Moscatel (MO) (Málaga D.O., Málaga) and cv. Zalema (ZA) (Condado de Huelva D.O., Huelva). The samples presented a reasonable range of variation for representing the winemaking areas classified as a semi-continental Mediterranean climate with short winters and long, dry and hot summers (the diurnal temperature can reach 40 °C).

Ripe grapes (MO, PX and ZA) were harvested and placed in single layers to sun dry for 10 days. After this period, the raisins (24° Bé of sugar content) were crushed and pressed and the residual grape pomace was used to obtain the overripe seeds, being manually separated. The separation procedure consisted of sifting the pomaces (the rest of skins, pulp and seeds) through a mesh (70 cm × 120 cm, approximately) that allowed the seeds to be quickly separated from the rest of the pomaces. Once the seeds were separated through the mesh, they were manually cleaned from the small rest of solid parts.

For each variety, 10 samples were taken from a global amount of about 10–15 kg of grape pomace. In turn, each sample consisted of 10 randomly selected seeds manually separated from the pomace.

Another set consisting of 3 kg of seeds (cv. Zalema) was selected to monitor the evolution of the phenolic composition during 16 days of the overripening process. In this case, the ZA variety was chosen as a representative white grape variety cultivated in southern Spain. Samples (100 g of seeds) were taken every day.

The samples were first analyzed by spectroscopic measurements and then subjected to phenolic extraction and a rapid resolution liquid chromatography (RRLC) analysis.

### 2.2. Instrumental Raman Analysis and Data Collection

Overripe seeds were crushed using an analytical mill (IKA, Staufen, Germany) and a manual mortar to homogenize the samples. Samples were then measured by using a BRAM-HR-1064 dispersive spectrometer (BaySpec Inc., San Jose CA, USA). The instrument provides laser excitation at 1064 nm, which is not the most popular wavelength because the Raman signal is inversely proportional to the fourth power of the laser wavelength. However, this long excitation wavelength allows the avoidance of fluorescence effects (common in vegetal samples due to some compounds as chlorophylls), which could mask the weak Raman signal.

Three spectrometers, used sequentially, allowed the scanning of all Raman shifts ranging from 300 to 3200 cm^−1^ at 2 cm^−1^ steps. A thermoelectric-cooled InGaAs array set at −55 °C served as a detector. Each spectral channel was integrated over a bandwidth of 4 cm^−1^.

The laser power was 450 mW (MiniLite-1064, BaySpec, CA, USA); its light was delivered to the Raman probe via a 105 μm optical fiber and then collimated by a lens. The laser line was cleaned by a narrow band-pass 1064 nm laser clean-up filter (Semrock, NY, USA). After passing a dichroic beam splitter (Semrock, NY, USA), the light was focused by a lens (f = 6 mm) onto the sample, also used to collect the back-scattered light. Raman signals (wavelengths longer than 1064 nm) were reflected by the dichroic beam splitter and further cleaned by a long-pass filter before being coupled into a 200 μm optical filter and sent back to the spectrometer.

The laser power was limited to 300 mW to avoid sample damage. In addition, the laser was slightly defocused on the sample surface in order to reduce the influence of the surface roughness of the grape seeds. The integration time was set at 20 s and three readouts were averaged during each scan. The samples were analyzed inside a 4 mL glass vial and measured eight times, rotating the vial after each scan. The vial was inserted into a suitable holder that was butt-coupled to the micro-optic unit. The entire instrument was portable and interfaced with a laptop PC that included dedicated BaySpec software for the management of hardware options and for spectra acquisition, display and first processing.

### 2.3. Phenolic Extraction and Determination by RRLC Analysis

Conventional techniques were applied to obtain the reference compositional data to explore the correlation with the Raman spectra.

The extraction and determination of the phenolic composition of overripe seeds were performed in triplicate according to the methodology described by Jara-Palacios et al. [10]. Overripe seeds were individually freeze-dried in a lyophilizer (Cryodos-80, Telstar Varian DS 102, Tarrasa, Spain) and subsequently ground to powder. An amount of 1 g of the overripe seed powder of each variety (PX, MO and ZA) was individually extracted with 5 mL of 75% methanol, shaking for 1 h in an incubating minishaker (VWR International, Barcelona, Spain). The supernatants were then centrifuged (4190 g, 10 min). The residue was twice submitted to the same process and the supernatants were combined.

The determination of flavan-3-ols (monomeric and procyanidins) as well as the hydroxycinnamic and benzoic acids of the overripe seed extracts were determined in triplicate according to the method of Jara-Palacios et al. [24] by rapid resolution liquid chromatography (RRLC). The chromatographic system was an Agilent 1290 (Agilent Technologies, Palo Alto, CA, USA) with a quaternary pump, UV-vis diode-array detector, an automatic injector and ChemStation software (Agilent Technologies, Palo Alto, CA, USA) using a C18 Poroshell 120 column (2.7 µm, 5 cm x 4.6 mm). The volume of injection was 0.5 µL.

The solvents were formic acid and water (1:999 mL:mL) as solvent A and acetonitrile as solvent B at the following gradients: 0–5 min of 5% B linear, 5–20 min of 50% B linear and 20–25 min of washing, which was followed by a re-equilibration of the column. The flow rate was 1.5 mL/min and the column temperature was set to 25 °C.

The identification of phenolics was performed according to the retention times of the standards (when available) and UV-vis spectra. Detection was also performed in an API 3200 Qtrap (Applied Biosystems, Darmstadt, Germany) equipped with an electrospray ionization source and a triple quadrupole-ion trap mass analyzer as described by Jara-Palacios et al. [24].

The quantification was made at 280 nm (flavanols and benzoic acids) and 320 nm (hydroxycinnamic acid acids) by comparing the areas and the retention times with the gallic acid, catechin and procyanidins B1 and B2 standards purchased from Sigma–Aldrich (Madrid, Spain). Total flavanols, total procyanidins, total oligomeric flavanols (trimers and tetramers) and total phenolic acids were estimated by summing the concentration of each individual phenolic and the results were expressed as mg/100 g of dry seeds (DS).

### 2.4. Chemometric Analysis

In the first phase, Raman spectra were smoothed using a five-point mobile average iterated five times. They were corrected for the baseline, which was evaluated by a linear least square fit between 2120 and 2820 cm^−1^ where no Raman bands occurred. The whole 500–2000 cm^−1^ range was selected at first for analysis. However, the granular aspect of the sample surface caused strong fluctuations of the background intensity, hampering the extraction of informative spectral features. Therefore, a further pre-treatment stage was introduced. The analysis was restricted to a 1240–1850 cm^−1^ spectral range where the strongest Raman peaks were located and where the background spectrum could be satisfactorily estimated by a three-order polynomial fit. The 1240–1300 cm^−1^ and 1800–1850 cm^−1^ spectral ranges were used to fit the polynomial, which was subtracted from the raw spectrum.

Processed spectra were analyzed by a principal component analysis (PCA), a common tool for data compression.

All processes related to image and spectral analyses as well as multivariate statistical treatments (principal components analysis) were performed with the software MATLAB R2011b (The Mathworks, Natick, MA, USA).

## 3. Results and Discussion

### 3.1. Raman Spectra and Data Processing of Overripe Seeds Varieties

**Figure 1:**
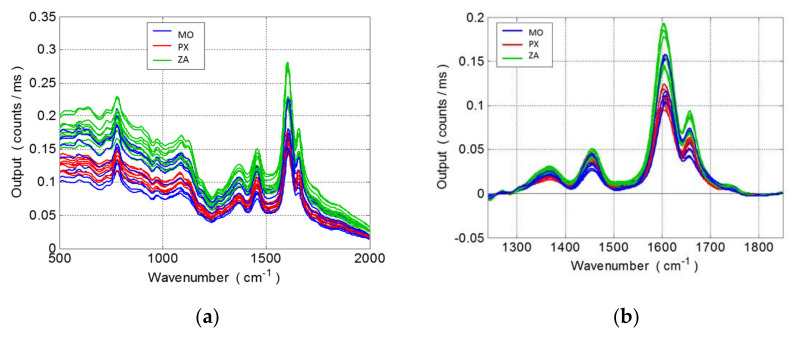
(**a**) Raw Raman spectra (500–2000 cm^−1^) of the entire grape seed collection from white grape varieties submitted to 10 days of postharvest sun drying (MO: Moscatel, PX: Pedro Ximénez, ZA: Zalema), (**b**) Raman spectra (1240–1850 cm^−1^) after the baseline correction according to the grape variety.

The raw Raman spectra (Figure 1a) of overripe seeds from the three grape varieties (MO, PX and ZA) submitted to 10 days’ postharvest sun drying were corrected for the baseline and the fully processed spectra (1240–1850 cm^−1^ spectral range) and are shown in Figure 1b. The corrected spectra were characterized by an intense band at 1600 cm^−1^, a medium band at 1650 cm^−1^ and two weak bands at 1360 and 1450 cm^−1^. Although the Raman spectra pattern was similar for the three grape varieties, a few differences among them could be observed for the Raman intensity mainly at 1600 and 1650 cm^−1^ peaks. According to the literature, the main functional groups assigned to the different vibrations present in the Raman spectra of grape seed samples are polysaccharides, lignin, fatty acids and phenolic compounds [22]. In particular, the band at 1655 cm^−1^ was assigned to the C-C stretch in lignin and fatty acids while the band at 1609 cm^−1^ to aromatic C=C skeletal stretching in phenolics.

**Figure 2:**
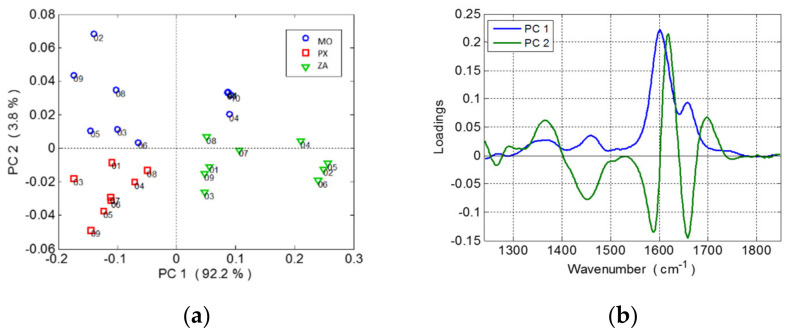
(**a**) Score plots of the principal component analysis (PCA) (first two principal components) for clustering grape seeds according to the grape variety (MO: Moscatel, PX: Pedro Ximénez, ZA: Zalema). (**b**) PC1 and PC2 loading plots.

The Raman spectra were compressed using a PCA. Three outliers (two of PX and one of ZA) had heavily distorted spectra and were removed from dataset. A new PCA model was then calculated with the remaining samples. Only the first four PCA explained more than 1% of the variance and the higher-order components were discarded. The first two components showed a discrimination ability between cultivars (explaining 96% of the total variance).Figure 2 shows the results of the PCA processing (the scatterplot of PC1 vs. PC2 (Figure 2a) and the spectra of the corresponding loading coefficients (Figure 2b)).

PC1 (92%) was related to the intensity of the main Raman spectrum. Overripe seeds of the ZA variety had positive scores (more intense spectra) along PC1 while those of PX had negative ones. Therefore, PC1 could split quite successfully the overripe seeds of ZA from those of the PX variety but not from MO, which had a stronger variance along this axis. On the other hand, PC2 (4%) explained the contrast between the intensity at 1620 cm^−1^ from one side and those at 1590 and 1660 cm^−1^. Therefore, it was related to weaker variations in the band fine structure. PC2 was able to separate the overripe seeds of MO (positive scores) from those of the PX and ZA varieties (negatives scores).

The mean values of the phenolic composition (mg/100 g DS) determined in overripe seeds according to the grape variety is shown in Table 1.

Quantitative differences were found for most of the individual compounds identified. The overripe seeds from the MO and ZA varieties were the richest sources of phenolics (281 and 179 mg/100 g DS, respectively) mainly due to the highest proportions of total flavanols (70% and 60%, respectively). Overripe seeds from MO had the highest contents of monomeric flavanols particularly while those from ZA were richest in procyanidins and oligomeric flavanols. In comparison, the overripe seeds from PX were poorer sources of phenolic compounds, indicating a significantly lower phenolic potential. The differences found in the total phenolic content with respect to the overripe seeds from the MO variety (two-fold higher) agreed with the results reported in a previous work [25].

In general terms, the compositional variability among the overripe seeds agreed with the differences observed in the intensity of specific peaks of the Raman spectra related to the presence of phenolic compounds (around 1600 cm^−1^) according to Nogales et al. [22].

### 3.2. Monitoring the Grape Seed Overripening Process by Raman Spectroscopy

**Figure 3:**
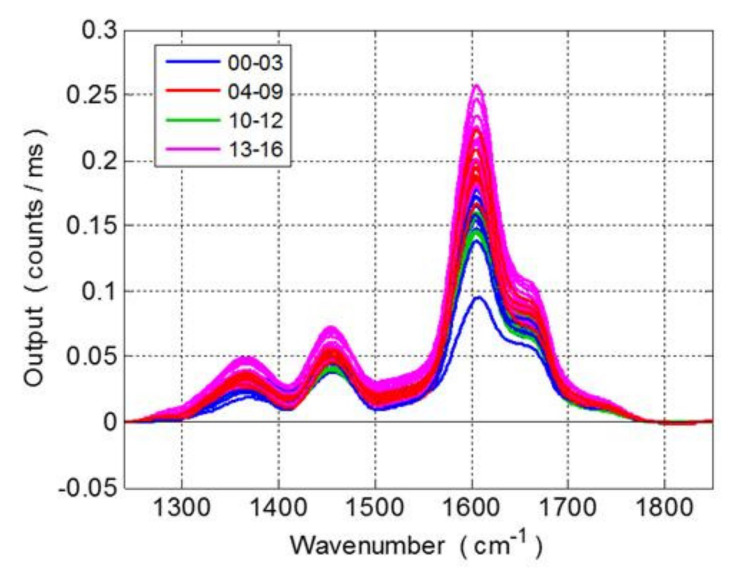
Baseline and corrected Raman spectra (1240–1850 cm^−1^) of the entire ZA grape seed collection during the overripening process (16 days of postharvest sun drying).

Figure 3 shows the fully processed Raman spectra of ZA grape seeds during the overripening process (16 days of postharvest sun drying). The Raman raw data were slightly noisy and, in a few cases, showed sensible distortion due to the irregularity of the sample surface. These, therefore, needed spectral pre-treatments. Principal component analysis (PCA) and Hotelling T-square statistics were applied to raw spectra for the detection and removal of irregular spectra with 36 outliers from 447 spectra being rejected (the confidence level was 95%). The remaining spectra were smoothed by a five-point mobile average, iterated five times and selected for further processing. As in the previous analysis, the spectral range of 1240–1850 cm^−1^ was then selected, and a background correction was applied by means of a three-order polynomial fit.

In general, the average intensity of the Raman band (with a higher load at 1600 cm^−1^) tended to increase in the first eight days of the overripening process then decreased and increased again from the thirteenth day onward. This spectral matrix was processed by a multivariate analysis aimed at correlating the spectroscopic data with the chemical information of grape seeds during overripening.

**Figure 4:**
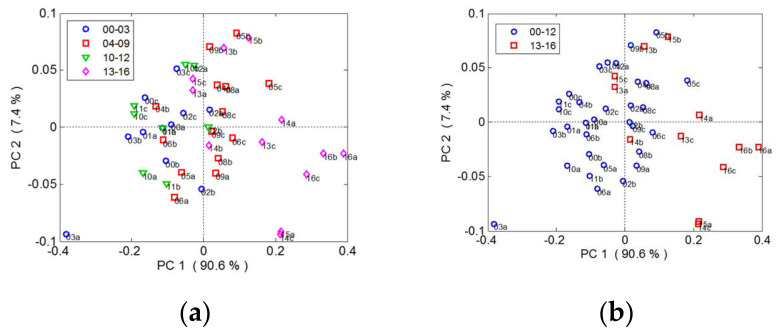
(**a**) Score plots of the PCA (first two principal components) for clustering ZA grape seeds according to four overripeness stages during 16 days of postharvest sun drying. (**b**) Score plots for clustering ZA grape seeds according to two overripeness stages.

A principal component analysis (PCA) was applied with the aim of monitoring the overripeness stages of grape seeds from their Raman spectral features, which could provide useful information in selecting the optimum date for wine production or for the recovery of high added value compounds from grape byproducts [26]. Figure 4 shows the PCA score plot in the PC1-PC2 subspace (accounting for 98% of the total variance) and the plot of the loadings. The PC1 (90.6%) was again related to the overall intensity of the band (higher loads around 1600 cm^−1^), which roughly followed the evolution of overripening although not monotonically. Tentatively, the sun drying period was divided into four stages (0–3, 4–9, 10–12 and 13–16 days). Nevertheless, a few steps overlapped (Figure 4a) and samples belonging to 10–12 days did not follow the general trend. Hence, a more satisfactory asymmetric two-group separation (Figure 4b) was found that split seeds with 11–16 days of overripening from 0–10 days. This separation could be related to the phenolic composition of seeds because, as found by the RRLC analysis regarding the changes during sun drying, the content of gallic acid tended to increase during the whole process and was more abundant in grape seeds after 10 days of postharvest sun drying.

**Figure 5:**
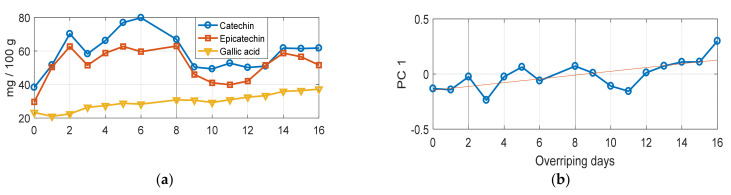
(**a**) Evolution of the major phenolic compounds identified in ZA grape seeds during the overripeness process (16 days of postharvest sun drying). (**b**) Temporal behavior of daily means for the PC1 score. All variables were auto-scaled to allow a comparison of trends.

Figure 5a shows the evolution of the major compounds identified in ZA grape seeds with (+)-catechin and (-)-epicatechin as the most abundant phenols followed by gallic acid. The daily mean behavior of (+)-catechin and (-)-epicatechin was quite similar and was more abundant in grape seeds with a lower degree of sun drying (0–10 days of postharvest sun drying). However, (+)-catechin had a clear maximum between the fifth and sixth day while (-)-epicatechin was between the sixth and ninth day. In comparison, the content of gallic acid tended to increase during the whole process and was more abundant in grape seeds with a higher degree of sun drying.

In order to compare the trends of PC1 with the concentration of major phenolics they were all auto-scaled by subtracting the mean and dividing by the standard deviation and are represented in Figure 5b. It can be observed that during the first 8–9 days, PC1 showed a generally increasing trend such as gallic acid but also strong fluctuations that were roughly synchronous with those of (+)-catechin and (-)-epicatechin, at least until day 15. The two strong minima at four and eleven days are noteworthy.

In order to understand the PC1 trend and the content of the main phenolic compounds, Pearson correlation coefficients were calculated between the daily means of PC1 scores and those of phenolic concentrations. They were below 0.4 for catechin and epicatechin, while gallic acid scored a more interesting 0.73.

## 4. Conclusions

Overripe seeds from sun-dried grapes were successfully separated according to the grape variety based on their specific dispersive Raman spectroscopy excited at 1064 nm, which was used to avoid natural sample fluorescence. Furthermore, the spectral results allowed the monitoring of different stages during the overripening process, which seemed to be related to the phenolic compound evolution. This technique appeared to have a good potential as a modern analytical tool for the evaluation of the postharvest sun drying procedure in grape seeds. The application of a multivariate analysis allowed the correlation of the spectroscopic data with the chemical information of grape seeds during the aforesaid procedure in order to better understand the applied procedure and implications in the chemical composition of the grape seeds. However, further studies should be developed with the aim of compressively optimizing the technique for predicting important nutraceutical indicators such as phenolics in grape byproducts.

## Figures and Tables

**Table 1:** Phenolic composition (mean values ± SD, *n* = 10) of overripe seeds according to grape variety (MO: Moscatel, PX: Pedro Ximénez, ZA: Zalema) determined by rapid resolution liquid chromatography/mass spectrometry (RRLC/MS).

	Grape Varieties		
Phenolic Compounds	MO	PX	ZA
Sum of benzoic acids	80.78 ± 5.01 ^a^	55.20 ± 1.37 ^b^	62.21 ± 1.98 ^c^
Sum of monomeric flavanols	189.85 ± 17.16 ^a^	46.32 ± 6.14 ^b^	105.00 ± 8.67 ^c^
Sum of procyanidins	7.26 ± 2.39 ^a^	5.28 ± 0.73 ^b^	8.21 ± 0.88 ^a^
Sum of oligomeric flavanols	2.94 ± 1.17 ^a^	1.60 ± 0.13 ^b^	3.46 ± 0.36 ^a^
Sum of total phenolics	280.85 ± 20.43 ^a^	108.41 ± 7.16 ^b^	178.89 ± 11.44 ^c^
Benzoic acids (280 nm)			
Gallic acid	47.21 ± 5.22 ^a^	22.13 ± 1.23 ^b^	30.27 ± 1.40 ^c^
Protocatechuic acid	16.13 ± 0.49 ^a^	15.22 ± 0.27 ^b^	15.17 ± 0.52 ^b^
Protocatechuic acid derivative	17.45 ± 1.79 ^a^	17.83 ± 0.46 ^a^	16.77 ± 0.97 ^a^
Monomeric flavanols (280 nm)			
(+)-Catechin	105.18 ± 15.15 ^a^	24.75 ± 4.47 ^b^	57.48 ± 3.93 ^c^
(-)-Epicatechin	84.66 ± 10.53 ^a^	21.56 ± 2.31 ^b^	47.51 ± 6.14 ^c^
Procyanidins (280 nm)			
Procyanidin B1	2.23 ± 1.42 ^a^	1.86 ± 0.41 ^a^	3.71 ± 0.46 ^b^
Procyanidin B2	1.45 ± 0.48 ^a^	0.99 ± 0.28 ^b^	1.83 ± 0.25 ^a^
Procyanidin B2 3-O-gallate	0.72 ± 0.11 ^a^	0.66 ± 0.04 ^a^	0.93 ± 0.10 ^b^
Procyanidin B7	0.86 ± 0.22 ^a^	0.67 ± 0.05 ^b^	0.90 ± 0.09 ^a^
Procyanidin EC gallate	1.98 ± 0.99 ^a^	1.09 ± 0.17 ^b^	0.75 ± 0.05 ^b^
Oligomeric flavanols (280 nm)			
Trimer	1.31 ± 0.40 ^a^	0.93 ± 0.12 ^b^	1.03 ± 0.08 ^ab^
Tetramer	1.64 ± 0.83 ^a^	0.68 ± 0.05 ^b^	2.43 ± 0.30 ^c^

Different letters in the same row indicate statistical differences (*p* < 0.05; ANOVA, Tukey test).

## Data Availability

Data are contained within this article.
